# Activated Mast Cells Combined with NRF2 Predict Prognosis for Esophageal Cancer

**DOI:** 10.1155/2023/4211885

**Published:** 2023-01-04

**Authors:** Xinxin Guo, Weitao Shen, Mingjun Sun, Junjie Lv, Ran Liu

**Affiliations:** ^1^Key Laboratory of Environmental Medicine Engineering, Ministry of Education, School of Public Health, Southeast University, Nanjing 210009, China; ^2^Cancer Institute of Fudan University, Fudan University, Shanghai 200032, China

## Abstract

**Background:**

Esophageal cancer (EC) had the sixth-highest mortality rate of all cancers due to its poor prognosis. Immune cells and mutation genes influenced the prognosis of EC, but their combined effect on predicting EC prognosis was unknown. In this study, we comprehensively analyzed the immune cell infiltration (ICI) and mutation genes and their combined effects for predicting prognosis in EC.

**Methods:**

The CIBERSORT and ESTIMATE algorithms were used to analyse the ICI scape based on the TCGA and GEO databases. EC tissues and pathologic sections from Huai'an, China, were used to verify the key immune cells and mutation genes and their interactions.

**Results:**

Stromal/immune score patterns and ICI/gene had no statistical significance in overall survival (OS) (*p* > 0.05). The combination of ICI and tumor mutation burden (TMB) showed that the high TMB and high ICI score group had the shortest OS (*p* = 0.004). We recognized that the key mutation gene NRF2 was significantly different in the high/low ICI score subgroups (*p* = 0.002) and positivity with mast cells (MCs) (*p* < 0.05). Through experimental validation, we found that the MCs and activated mast cells (AC-MCs) were more infiltration in stage II/III (*p* = 0.032; *p* = 0.013) of EC patients and that NRF2 expression was upregulated in EC (*p* = 0.045). AC-MCs combined with NRF2 had a poor prognosis, according to survival analysis (*p* = 0.056) and interactive analysis (*p* = 0.032).

**Conclusions:**

We presume that NRF2 combined with AC-MCs could be a marker to predict prognosis and could influence immunotherapy through regulating PD-L1 in the EC.

## 1. Introduction

With the rapid growth and aging of the world's population, cancer will be the main reason for the rising burden of disease in the 21^st^ century. Esophageal cancer (EC) is the sixth leading cause and has the eighth highest incidence rate in the world. In China, 90% of EC is esophageal squamous cell carcinoma (ESCC), and esophageal adenocarcinoma (EAC) is more common in western countries [1]. Traditional technologies such as radiotherapy, chemotherapy, surgery, and trimodality are the main therapy methods for EC [2], but the five-year survival rate is still less than 15% [3]. Hence, many researches were aimed to finding meaningful therapeutic and prognostic biomarkers for EC in order to improve the prognosis and prolong the lives of patients.

Recently, immunotherapy had been proven to have prospective results for EC therapy; however, the immunotherapy's effectiveness was affected by the complex tumor microenvironment (TME), so not all patients are benefited from these therapeutic interventions [4]. The majority of research studies indicated that tumor-associated immune cells, especially innate immune cells such as macrophages, dendritic cells, and mast cells (MCs), were related to immunotherapy and tumoral responses [5–7]. MCs were bone marrow-derived cells which could be recruited into tumor tissue by SCF, chemokine factors, and so on. Hypoxia, the accumulation of (lactic acid, adenosine, PGE_2_, IFN-*γ*, etc.) and low pH in TME could activate MCs discharge particles to pro- and antitumoral by IgE/Fc*ε*RI pathway [8–10]. Activated mast cells (AC-MCs) have been recognized as an important prognostic indicator and immune therapy target for cancers [11].

The prognosis was affected by the complex immune cell infiltration (ICI) in TME. Recently, some researchers created models according to immune cells and differential expression genes (DEGs) to predict prognosis. Apart from this, somatic mutation genes also influenced a patient's prognosis and immunotherapy response [12]. TP53 mutations affected the immunophenotype in gastric cancer and influenced the patient's prognosis [13]. In addition, some clinical trials also indicated that KEAP1/NRF2 mutations can be regarded as predictive markers for immunotherapy and prognosis makers for cancer [14]. Tumor mutation burden (TMB) is defined as the total number of somatic gene coding errors, base substitutions, gene insertions, or gene deletions detected per million bases. Some research studies suggest that TMB is associated with the emergence of neoantigens which trigger antitumor immunity [15, 16]. Tumor patients with higher TMB had higher survival rates [17, 18]. A few somatic mutations in tumor DNA can be translated into neoantigens, which could be present on the surface of cells in the form of the major histocompatibility complex and recognized by the immune system [19]. However, the combined effects of ICI and TMB in predicting prognosis remained unknown.

In this study, we established multiple immune score models and TMB to predict prognosis and immunotherapy. Our results indicated that the combined immune score with TMB was related to prognosis and PD-L1, and we recognized the key mutation gene NRF2. We also found that NRF2 was related to AC-MCs. Based on these results, we analyzed the combined effects of NRF2 and AC-MCs for prognosis by TCGA database and experiment verification. Our results showed that there is an interaction between NRF2 and MCs, especially the higher NRF2, which had a worse survival rate. In total, we thought NRF2 combined with AC-MCs could be used to predict the prognosis for EC and provide a new direction for the prognostic study of esophageal cancer.

## 2. Materials and Methods

### 2.1. EC Datasets and Samples

A total of 524 EC samples were downloaded from the TCGA-GDC database (https://portal.gdc.cancer.gov/) and the GEO database (https://www.ncbi.nlm.nih.gov/geo/). The RNA sequencing (RNA-seq; fragments per kilobase million value) data and the clinical information (BCR-XML) including futime, survival state, age, gender, grade, stage, and the TNM stage system were downloaded from TCGA-EC. The microarray data (GSE68698, GSE69925, and GSE161533) were downloaded from the GEO. To increase the readability of the data, the FPKM values were transformed into TPMs (transcripts per kilobase million), which were identical to the results of microarrays, and clinical information (BCR-XML) was transformed into a matrix. The “limma” R package and the “sva” R package were used to merge the RNA array. The “ComBat” algorithm was used to decrease the likelihood of batch effects from different biological and technical biases between different datasets. Because the clinical information in GEO is limited, we only use the clinical features from TCGA when analyzing the results, which refer to the clinical information. In addition, we collected 33 ESCC patients' tissues and 30 ESCC pathological sections who had not received therapy from Huai′an First Hospital, in 2021. The detailed information about the patient is listed in Table 1. This study was performed in accordance with the principles of the Helsinki Declaration and was performed, reviewed, and approved by the Ethics Committee of Zhongda Hospital of Southeast University; the grant number is 2021ZDKYSB004.

### 2.2. Estimation of Stromal and Immune Scores

The “CIBERSORT” algorithm is a deconvolution algorithm and was used to quantify the infiltration level of the distinct immune cells based on the input reference gene sets and repeated 1000 times to ensure stability. The “ESTIMATE” algorithm was used to calculate the immune scores, stromal scores, and estimate scores by the “estimate” R package. At the same time, we analyzed the prognostic value of immune stores and stromal scores and their relationship with clinical features.

### 2.3. ICI Clusters

We used the R packages “biomanager” and “consensus” to divide the samples into different clusters according to the immune cells' relative fraction levels in EC. And the prognostic values in different ICI groups were indicated by the “survival” and “survminer” R packages. The immune cells in the differnt clusters were reshaped by “ggpubr” package. Results were visualized through heat maps by the “pheatmap” R package.

### 2.4. DEGs Associated with the ICI Phenotype and Gene Clusters

DEGs in different ICI clusters were determined by setting the significance cutoff to *p* < 0.05 (adjust) and logFC >1, which was performed by the “limma” R package. According to DEGs, the samples were divided into different types using the “biomanager” and “consensus cluster plus” R packages. Immune cells in different gene clusters were analyzed by “ggpubr.” We also analyzed the prognostic value of different gene clusters as indicated by the “survival” and “survminer” R packages.

### 2.5. ICI Scores

First, unsupervised clustering was used to deal with the samples in TCGA and GEO according to DEG values, which were positively or negatively correlated with the cluster signature and described as ICI gene signatures A and B, respectively. Second, the “Boruta” R package was used for dimension reduction of the ICI gene signatures A and B and to extract feature genes. Third, principal component 1 was extracted as the signature score by using the principal component analysis (PCA). Finally, the formula that defined the ICI score of each patient was ICIscore=∑*PC*1*A* − ∑*PC*1*B*, and we divided the ICI score into a high ICI score group and a low ICI score group. According to the ICI score, the functional enrichment analyses of GO and KEGG pathways were analyzed using the “clusterProfiler” R package for the feature genes in the high ICI score group and the low ICI score group. In order to know the prognostic significance of the ICI score, we also analyze the connection between clinical features and ICI score based on the TCGA database.

### 2.6. Somatic Alteration Data Analysis

The related somatic mutation datasets for EC were downloaded from the TCGA-GDC database. Tumor mutation burden (TMB) is defined as the total number of somatic coding errors in genes, base substitutions, and gene insertion and deletion errors in EC. The “ggpubr” R package was used to analyze the TMB for high ICI scores and low ICI scores. The mutation genes with high ICI scores and low ICI scores were identified through the “maftool” R package, and the top 30 genes with the highest mutation frequency were listed.

### 2.7. Toluidine Blue Staining

Toluidine blue staining was used to detect the number and distribution of MCs in ESCC. Paraffin-embedded tissues were dewaxed in different concentrations of alcohol, subsequently stained with toluidine blue (Solarbio, China) for 15 min, and washed with PBS 3 times. Photomicrographs of ten fields were taken at different magnifications using the camera (ZEISS, Germany), and the mean value was used to describe the number and distribution of MCs in EC. The AC-MCs rate was calculated by the ratio of the AC-MCs number to the total MCs number.

### 2.8. Q-PCR and Western Blot Analyzed the Expression of MCs Related Genes and NRF2

We analyzed the relative genes in ESCC tumor tissue and para-tumor tissue. Trizol was used to extract RNA from tumor tissue and para-tumor tissue. The RAN was cDNA obtained by reverse transcription according to the protocol (Vazyme, China). SYBR green was used to complete the related expression. The Q-PCR procedure followed the protocol (Vazyme, China). The primer sequences were as follows: 95°C 3 min, 95°C 30 s, 60°C 15 s, 72°C, 30 s for 40 cycles, and solubility curve. The primer sequence is listed in Table S1. In addition, tissues were addedto RIPA and lysed in an ultrasound machine. After being divided by SDS-PAGE, the proteins were transferred onto PVDF membranes and then blocked with 5% skim milk for 2 h, subsequently incubated with primary antibodies of NRF2 (1 : 1000), TPSB2 (1 : 1000), and GAPDH (1 : 5000) overnight at 4°C and next incubated with secondary antibodies for 1 h at room temperature. The target protein was visualized by the ECL Gel Image System and analyzed by the software Image J.

### 2.9. Statistical Analysis

All statistical analyses were accomplished with R version 4.0.3, GraphPad Prism 8, and SPSS version 25.0. The comparison between the two groups was tested by the Wilcoxon test and *T* test; otherwise, it was tested by Kruskal–Wallis H and ANOVA. The survival curves for the subtypes were accomplished with the Kaplan–Meier plotter. The chi-square test was used to analyze the correction between the ICI score subtypes and somatic mutation frequency. The chi-square test was used to analyze the classified variable. And the correlation analysis was completed by Pearson's analysis. Univariate and multivariate Cox regression models were used to analyze the prognosis. The interaction of NRF2 and AC-MC was analyzed by interactive analysis. All analyses were two-tailed, and *p* < 0.05 was regarded as the statistically significant level.

## 3. Results

### 3.1. The Characteristic of ICI in the TME of EC

**Figure 1:**
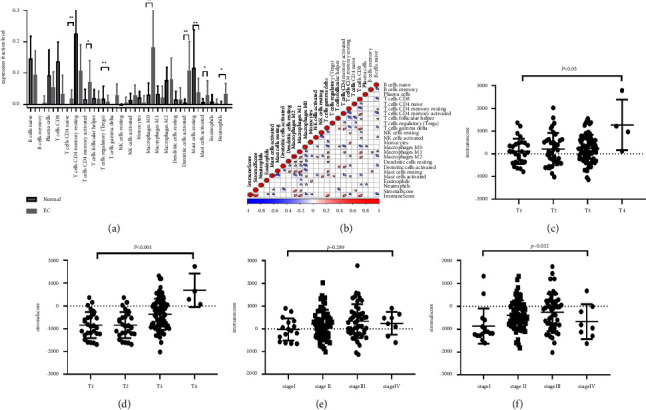
The landscape of ICI in the TME of EC. (a) The immune cells in EC tissue and para-cancer tissue. (b) The landscape of 22 immune cells' interactions in TME. (c and d) Association of immune scores with *T* stage (c) and clinical stage (d). (e and f) Association of stromal scores with *T* stage (e) and clinical stage (f). ^*∗*^*p* < 0.05; ^*∗∗*^*p* < 0.01; ^*∗∗∗*^*p* < 0.001.

22 human immune cells were calculated through the CIBERSORT algorithm according to the TCGA and GEO databases and found to have differential expression in tumor tissues and para-carcinoma tissues. These results suggested that the relative fractions of Tregs and resting MCs in the tumor tissue were lower than those in para-carcinoma tissue, but the naive CD4 *T* cells, activated CD4 memory T cells, M0 macrophages, activated DCs, activated MCs, and neutrophils in the tumor tissues were higher compared with para-carcinoma tissues (Figure 1(a)). The “corrplot” R package was used to generate a correlation coefficient heatmap to visualize the landscape of 22 immune cells' interactions in TME (Figure 1(b)). Additionally, the ESTIMATE algorithm was used to calculate the immune scores and stromal scores according to the levels of immune cells in EC. According to the clinical information from the TCGA database, we explored the relationship between clinical features and estimate scores. These results suggested that the immune scores and stromal scores were not associated with survival time, but clinical stage and *T* stage were related to stromal scores, and *T* stage was related to immune scores (Figures 1(c)–1(f)).

### 3.2. Different Patterns Were Used to Predict the Prognosis

**Figure 2:**
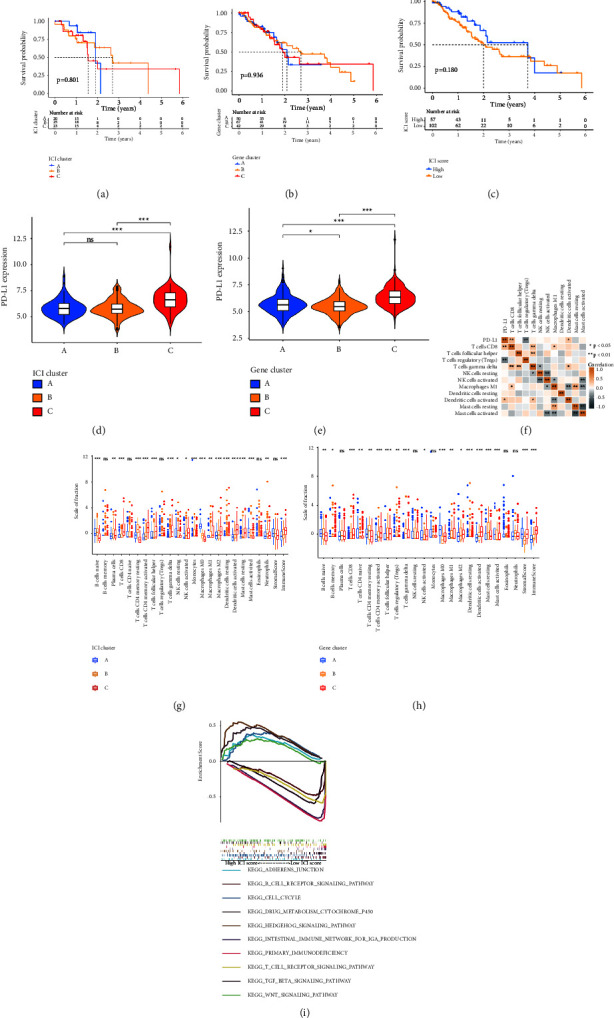
Different prognostic models constructed based on immune cells and DEGs. (a) Kaplan–Meier curves for overall survival of EC with different ICI clusters. (b) Kaplan–Meier curves for overall survival of EC with different gene clusters. (c) Kaplan–Meier curves for overall survival of EC with different ICI scores. (d) The expression of PD-LA in ICI clusters. (e) The expression of PD-L1 in ICI clusters. (f) The relationship between PD-L1 and immune cells. (g) The fraction of tumor immune cells in three ICI clusters. (h) The fraction of tumor immune cells in three gene clusters. (i) Enrichment plots showing signaling pathways in high/low ICI scores. ^*∗*^*p* < 0.05; ^*∗∗*^*p* < 0.01; ^*∗∗∗*^*p* < 0.001.

We analyzed the prognosis value of stromal scores and immune scores, but the results suggested that the score patterns were unrelated to prognosis (Figures S1(A) and S1(B)). So, we try to create new patterns according to immune cells and DEGs to predict the prognosis. First, the ICI types were divided into three clusters (Figure S1(C)). However, the three ICI clusters have no significant survival difference in EC (Figure 2(a)). Then, we constructed another subtype according to DEG (Figure S1(D)). Similarly, different gene clusters were unrelated to prognosis (Figure 2(b)). However, the ICI clusters and gene clusters were all related to PD-L1 (Figures 2(d) and 2(e)). So, we analyzed the immune cells and DEGs in the clusters, and we found that PD-L1 was more highly expressed in cluster C. The main immune cells in cluster C were CD8^+^*T* cells, CD4^+^*T* follicular helper cells, *T* cells gamma delta, NK cells, M1 macrophages, DCs, Tregs, and MCs (Figures 2(g) and 2(h)). At the same time, we also analyzed the relationship between the immune cells in cluster C and PD-L1, and the results suggested that PD-L1 is positively related to CD8^+^*T* cells and DCs but negatively related to Tregs (Figure 2(f)). The heatmap delineated the transcriptomic profile of all DEGs in three gene clusters and gene types (Figures S1E and S1F). To achieve quantitative indicators of the ICI landscape in EC patients, PCA was used to calculate two aggregate scores according to the ICI score A from ICI signature gene A and the ICI score B from ICI signature gene B (Table S2). In this research, the individual score of patients was computed through the ISA and ISB of each patient. All the patients were divided into two groups (high ICI score and low ICI score). We analyze the prognostic value of the ICI score. The survival rate in the two ICI score groups has no statistical difference (Figure 2(c)), but statistical analysis showed that survival status and the TN stage system were related to ICI score (Figures S2E–S2G). Meanwhile, we analyzed the main pathways in high ICI scores, such as adherens junction, cell cycle, Hedgehog signaling pathway, TGF-*β* signaling pathway, and Wnt signaling pathway, while the main pathways in the low ICI score were the *B* cell receptor signaling pathway, drug metabolism cytochrome P450, intestinal immune network for IgA production, primary immunodeficiency, and *T* cell receptor according to KEGG (Figure 2(i)). Functional enrichment analysis suggested that the main functions of the high ICI score group in the biological process were response to virus, type I interferon signal pathway, and response to tumor necrosis factor, but in the low ICI score group were extracellular matrix organization and endodermal cell differentiation. The main functions enriched in the cellular component of the high ICI score group were the extracellular matrix immunological synapse, membrane raft, anchored component of membrane, and apical plasma membrane, while in the low ICI score group, they were the endoplasmic reticulum lumen, extracellular matrix, and fibrillar collagen trimer (Figures S2A–S2D). These results suggested that the ICI score may be related to the prognosis of EC.

### 3.3. Combine ICI Score with TMB Predict Prognosis

**Figure 3:**
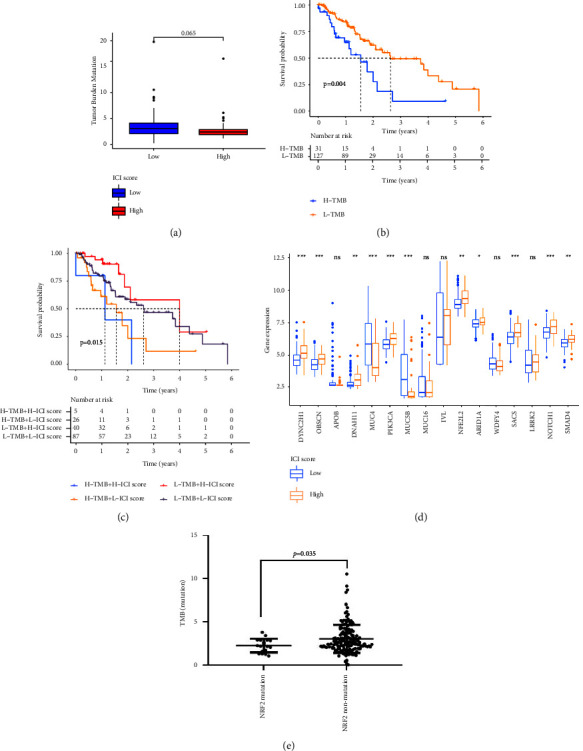
Interaction between the ICI score and the TMB. (a) TMB difference in the high ICI score and low ICI score. (b) Kaplan–Meier curves for high and low TMB groups of the TCGA-EC cohort. (c) Kaplan–Meier curves for patients in the TCGA-EC cohort stratified by both TMB and ICI scores. (d) The relative expression level in the high and low ICI score groups. (e) The value of TMB for NRF2 mutations and non-NRF2 mutations. ^*∗*^*p* < 0.05; ^*∗∗*^*p* < 0.01; ^*∗∗∗*^*p* < 0.001.

Most evidence indicated that TMB could be used to evaluate the predictive prognosis [20, 21]. In the study, we analyzed the relationship of TMB with the somatic mutation landscape in the EC and ICI scores, but the result showed that the TMB showed no significant differences between the two groups (Figure 3(a)). Then, we divided the samples into high/low TMB, and the result suggested that the survival rate of low TMB was higher than that of high TMB (Figure 3(b)). At the same time, when we combined the ICI score with the TMB, we found that the survival rate in the group with a low TMB and a low ICI score was the longest, whereas the group with a high TMB and a high ICI score was the shortest (Figure 3(c)). Furthermore, we assessed the distribution of somatic variants in EC driver genes between the high/low ICI score subgroups. The top 30 genes with the highest alteration frequency were further analyzed (Figures S3A and S3B). We also analyzed that the relative expression of mutation genes in high/low ICI score subgroups; DYNC2H1, OBSCN, DNAH11, PIK3CA, MUCSB, NRF2, ARID1A, SACS, LRRK2, NOTCH1, and SMAD4 were all significantly different between the high/low ICI score subgroups (Figure 3(d)). After analyzing the role of these genes in TMB, we found that NRF2 was related to TMB (Figure 3(e)). We further analyzed the prognosis value of NRF2 and indicated that the expression of NRF2 was related to the survival status, TNM system, and grade Figures S(3C-3F). However, univariate variables and multivariate Cox regression models were used to investigate the relationship between the NRF2 mutation and the overall survival of EC patients, and the result revealed that the NRF2 mutation was not an independent prognostic factor for OS in EC (Table S3).

### 3.4. Combining AC-MCs with NRF2 Could Predict Prognosis

**Figure 4:**
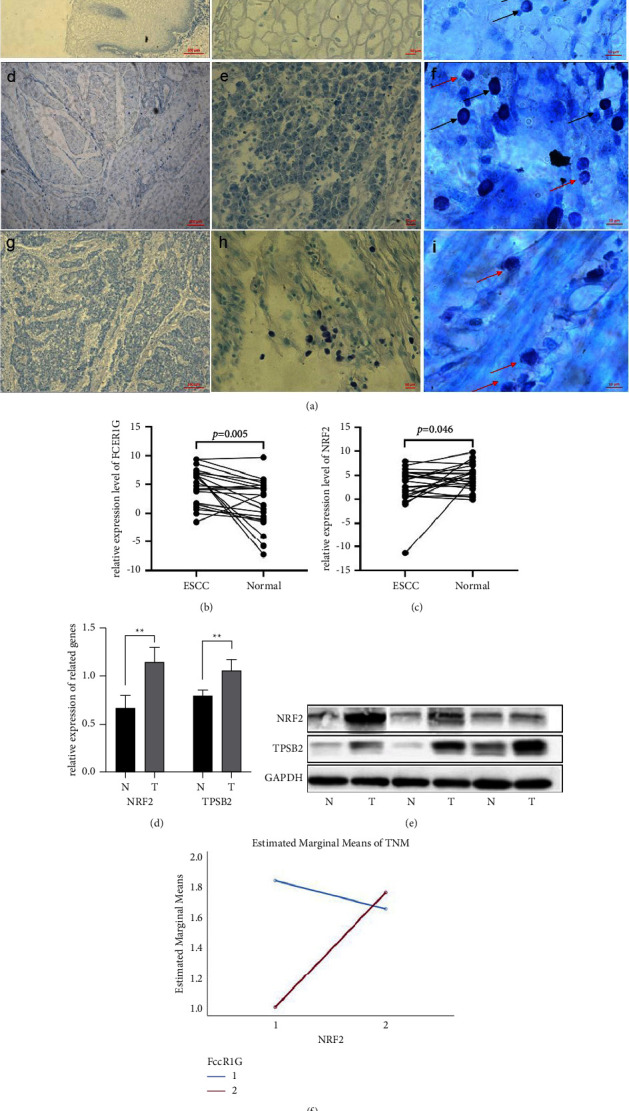
The infiltration of MCs in EC and the related gene expression as well as its relationships. (a) The MCs' infiltration of EC tissues. (A/B/C) Squamous epithelium. (D/E/F) Tumor nest. (G/H/I) Muscularis propria. The black arrow represents undegranulated MCs, and the red arrow represents granulated MCs. (b) Expression of FCER1G. (c) Expression of NRF2. (d) Statistical analysis of TPSB2 and NRF2 protein expression. (e) Protein expression levels of NRF2 and TPSB2. (f) Interaction of NRF2 and AC-MCs. ^*∗*^*p* < 0.05; ^*∗∗*^*p* < 0.01.

Considering the relationship between the TMB and ICI scores, what follows is the relationship between NRF2 and immune cells. We found that NRF2 was only related to MCs (Table S4). Next, we attempt to assess the combined effect of NRF2 and MCs for predicting prognosis in EC. We collected 30 EC patients' tissue slices and their clinical information to analyze the number and distribution of MCs/AC-MCs and their prognosis value. MCs were characterized by blue densely basophilic granules in the intracytoplasmic, and AC-MCswerecharacterized by many blue dye particles surrounding the cells. Our results showed that MCs were mainly in the muscular layer (*p* < 0.05) squamous epithelium (0.67 ± 3.46, Figure 4(a)-A/B/C), tumor nest (4.48 ± 9.63, Figure 4(a)-D/E/F), and muscularis propria (36.33 ± 37.84, Figure 4(a)-G/H/I). We also calculated the rate of AC-MCs in EC tissue. The MCs in patients in stage III were higher than those in patients in I and II (Table 1). We also analyzed the related gene, which could activate MCs. These results showed that Fc*ε*R1A (Figure 4(b), *p* < 0.005), NRF2 (Figure 4(b), *p* < 0.05), Fc*ε*R1G (Figure S4D, *p* < 0.000), and PD-L1(Figure S4E, *p* < 0.05) were all upregulated in tumor tissue (Figures S4D and S4E), and the protein level of NRF2 and TPSB2 was also higher expressed in tumor tissue (Figure 4(d)–E). The expression of NRF2 was related to Fc*ε*R1A (Figure S4E, *r* = 0.515), and PD-L1 was related to Fc*ε*R1G (Figure S4G, *r* = 0.468). We divided the expression of NRF2 and Fc*ε*R1G into two groups by median, and interaction analysis was used to explore the interaction of NRF2 and AC-MCs with TNM. Cox results suggested that NRF2 and MCs were all unrelated to OS (Figures S4A and S4B), but the group with low NRF2 and high Fc*ε*R1G was the lowest malignant (Figure S4C). Most importantly, there is an interaction between NRF2 and MC (Figure 4(f)). Hence, we thought that a combination of NRF2 and AC-MCs could be a prognosis maker for EC.

## 4. Discussion

The majority of studies have demonstrated that the heterogeneous TME and TMB participated in tumor progression, prognosis, and therapeutic for EC. However, clarifying the modulation of TME and TMB as well as their combination effects during EC remains a challenge. Our study comprehensively described the ICI landscape and somatic mutation gene landscape and constructed different patterns to quantify the ICI and TMB by the “CIBERSORT” and “ESTIMATE” algorithms to predict prognosis and the relationship with PD-L1. We found that the combined immune filtration cells and tumor mutation burden could predict the prognosis for EC. At the same time, we recognized the key mutation genes NRF2 and immune cells (mast cells), which played an important role in predicting prognosis. We verified the combined role of NRF2 and mast cells in EC patient and found that combined NRF2 and MCs would be a prognostic target and provide new insight into the prognosis of EC.

Multiple pieces of evidence have demonstrated that dysfunctional immune cells in the TME lead to immunosuppression and promote tumor survival and progression [22–24]. In this study, we analyzed the ICI landscape of EC according to the TCGA and GEO databases. Our results indicated that CD4 *T* cells, M0 macrophages, AC-MCs, and activated DCs were increased, but the Tregs and resting MCs were decreased in tumor tissue. Tregs suppress the activation and proliferation of multiple types of immunocompetent cells such as CD4^+^*T* cells, CD8^+^*T* cells, B cells, NK, and *T* cells, as well as suppressive immunoreaction [25]. CD4^+^*T* cells could increase the secretion of IL4, IL2 promoting breast cancer progression, and the mature dendritic cells induced the proliferation of CD4^+^*T* cells [26, 27]. AC-MCs could produce VEGF, PDGF, MMP9, and PGE2 to promote angiogenesis and tumor migration [28]. Moreover, AC-MCs' secreted cytokines could also influence the development and function of *T* cells and *B* cells [29]. Apart from evaluating the infiltration of single immune cells, we also attempt to quantify the ICI landscape to evaluate the prognosis through built-score patterns. In previous studies, the ESTIMATE algorithm has been used to analyze the immune scores and stromal scores, and it has been suggested that the risk model is beneficial for the early identification of high-risk patients to formulate an individualized treatment project and improve the possibility of an immunotherapy response [30, 31]. In our study, based on the stromal scores and immune scores, we divided the patients into high-score and low-score groups. We found that the survival probability in the two groups did not significantly change, but the stromal scores were higher in stage III, and the higher the immune scores and stromal scores, the higher the *T* stage. At the same time, we divided the samples into three parts based on the infiltrated immune cells. Our results demonstrated that the immune cells which have immunosuppressive function were focused on ICI cluster C. PD-L1, a key immune checkpoint, was higher in ICI cluster C. Previous evidence had shown that immune cell-related genes could predict disease progression and immunotherapeutic responses [32, 33]. Based on the immune-related gene in EC, we divided the patient into three ICI gene clusters. The results suggested that ICI gene cluster C had a more favorable immune-activated type with the highest density of CD8^+^*T* cells, M1 macrophages, activated DCs, and CD4^+^ T follicular helper cells [34–36]. Additionally, the expression of PD-L1 was highest in ICI gene cluster C. Hence, the patients in ICI gene cluster C might have a better immune response. The outcome of our analysis was in accordance with the previous study, which indicated that ICI clusters and ICI gene clusters in EC might influence the expression of PD-L1 [37].

In recent years, gene clusters related to immune response and proliferation were used to predict the outcome of cancers and identify high-risk patients; the distant metastasis-free survival in high-score immune gene was higher than low-score in breast cancer [38]. The prognosis value of the ICI score was calculated by the “Boruta algorithm” based on the immune cell-related gene, which has been proven in head and neck squamous cell carcinoma [39]. In the current study, we assessed the prognosis value of the ICI score in EC and found that there was no significant difference in OS in high/low ICI scores , but the ICI score was higher in alive, no lymph node metastasis samples. Through KEGG, our results indicated that the high ICI score mainly regulates the hedgehog signaling pathway, TGF-*β* signaling pathway, Wnt signaling pathway, and so on. Hedgehog signaling could be induced by activated *T* cells and NK cells and participate in immunotherapy [40]. Repression of the Wnt signaling pathway would decrease the expression of PD-L1 and increase the immune-killing effect of NK cells [41]. The TGF-*β*/EMT signaling pathway influenced the expression of PD-L1 and promoted immune escape. All these results demonstrated that the ICI score was related to the PD-L1 but was not an independent prognosis marker for EC [37].

The majority of studies demonstrated that TMB was related to prognosis and could be a marker for predicting the effectiveness of immune checkpoint inhibitors in cancer [42, 43]. Mutation genes related to TMB were crucial prognostic biomarkers for cancers [32, 44]. In our study, we analyzed the somatic mutation landscape according to TCGA. Our results indicated that the high TMB level had a poor OS, and the combination of the TMB with ICI scores showed that the high TMB and low ICI group had the worst OS. Meanwhile, these results indicated that TP53, TTN, MUC16, LRP1B, and SYNE1 were high-frequency mutations in EC. Especially, NRF2 was not only a high-frequency mutation gene in EC but also significantly different in ICI score groups. There was a study that reported that NRF2/KEAP1 mutations correlate with higher TMB value/PD-L1 expression and potentiate improved clinical outcomes with immunotherapy [45]. An NRF2 mutation could disrupt the weak binding of Keap1 with the NRF2-DLG motif and activate NRF2 to promote tumor progression [46]. Considering the complexity of mutations, we only detected the expression of NRF2 and the prognosis value in EC. In our result, the NRF2 was upregulated in EC, but not an independent prognostic biomarker, which was different from previous research studies [47]; the reason probably was that the number of patients was not enough. Meanwhile, we analyzed the relationship between NRF2 and immune cells and found that NRF2 was related to MCs. Other studies indicated that NRF2 could activate MCs, IgG/Fc*ε*RI promoted the phosphorylation of Lyn and activated Syk/PI3K, LAT/p38, and LAT/Raf-1/ERK1/2 pathways, and the AKT-Nrf2 and p38MAPK-Nrf2 signal pathways play an important role in hypersensitivity induced by MCs [48, 49]. Hence, we analyzed the MCs in EC tissue and found that MCs were irrelevant to OS. Surprisingly, the combination of NRF2 with MCs could affect prognosis. In addition, previous studies indicated that MCs could express PD-L1 and play a crucial role in immunosuppression [50]. Our results also indicated that Fc*ε*R1G could activate MCs, and the AC-MCs were positively related to PD-L1, but the mechanism by which activated MCs regulated PD-L1-induced immunosuppression deserves deep research. Therefore, we thought combining NRF2 with MCs would be used to predict prognosis. However, whether the NRF2-activated MCs are involved in immune suppression in EC needs further study.

In summary, we comprehensively analyzed the ICI landscape and TMB of EC and found that high ICI and high TMB had worse prognoses. We also recognized key mutation genes and immune cells and analyzed the common prognostic value of NRF2 with MCs by experiment verification and database analysis. Nevertheless, several limitations in our study should be considered. First, due to the limited patient information from TCGA, a larger sample size and sufficient information were required for further proof of our results. Second, the role of NRF2 and MCs participated in immune regulation and tumor progression in EC needs further experimental study. Third, it is not enough to clarify different patterns and MCs that could influence immunotherapeutic effectiveness only by analyzing the relationship with PD-L1. In all, we found that high ICI and high TMB could affect the prognosis, and the combination of NRF2 with AC-MCs had a worse prognosis and could be an effective prognostic factor for EC.

## Figures and Tables

**Table 1:** The relationship between MCs with clinicopathological features of ESCC.

Clinicopathological features	*N*	MC ($\bar{x}\pm s$)	*P*	*N*	Fc*ε*R1G ($\bar{x}\pm s$)	*P*
Gender						
Male	8	25.87 ± 15.22	0.299	21	2.09 ± 6.46	0.69
Female	13	33.07 ± 33.4681		4	1.04 ± 1.99	
Age						
≤65	16	24.37 ± 17.52	0.033^*∗*^	9	0.41 ± 0.61	0.15
>65	5	49.40 ± 46.39		16	2.77 ± 7.37	
Differentiation						
High differentiation	4	43.00 ± 11.91		5	0.24 ± 0.22	
Middle differentiation	10	32.10 ± 37.69	0.446	14	3.07 ± 7.87	0.568
Low differentiation	7	20.57 ± 13.22		6	0.64 ± 0.46	
*T*						
*T*1-*T*2	7	42.28 ± 42.13	0.160	7	1.24 ± 1.31	0.345
*T*3-*T*4	14	24.35 ± 16.29		8	2.19 ± 7.01	
*N*						
*N*0	13	20.23 ± 14.60	0.119	10	0.99 ± 1.15	0.179
*N*1–*N*3	8	46.75 ± 36.95		15	2.54 ± 7.67	
*M*						
*M*0	—	—	—	22	0.79 ± 0.97	<0.001^*∗*^
*M*1	—	—		3	10.24 ± 17.24	
Stage						
I	4	26.25 ± 19.25	0.032^*∗*^	—	—	
II	8	15.00 ± 10.46		12	1.05 ± 1.06	0.095
III	8	50.12 ± 34.36		13	2.72 ± 8.26	

## Data Availability

The datasets supporting our results are available in the TCGA and GEO database as well as data sources in the method. The data of our cohort are provided in tables, and further inquiries can be directed to the corresponding authors.

## Abbreviations

EC: Esophageal cancer
EAC: Esophageal adenocarcinoma
ESCC: Esophageal squamous cell carcinoma
TME: Tumor microenvironment
NK: Natural killer
DCs: Dendritic cells
Tregs: Regulatory *T* cells
MCs: Mast cells
AC-MCs: Activated mast cells
TCGA: The Cancer Genome Atlas database
GEO: Gene Expression Omnibus
ICI: Immune cell infiltration
TMB: Tumor mutation burden
NFE2L2/NRF2: Nuclear factor erythroid 2-related factor 2
RYR2: Ryanodine receptor 2
KEAP1: Kelch-like ECH-associated protein 1
TPMs: Transcripts per kilobase million
DEGs: Differentially expressed genes
PCA: Principal component analysis
KEGG: Kyoto Encyclopedia of Genes and Genomes
GO: Gene Ontology
ISA: ICI signature gene A
ISB: ICI signature gene B.

## Supplementary Materials

Supplementary Materials Table S1: the primer sequences of related genes. Table S2: the DEGs in ICI gene signatures A and B. Table S3: association with overall survival and clinicopathological characteristics in patients from the TCGA database using Cox regression. Table S4: correlation of NRF2 with immune cells in patients from the TCGA database and GEO database using the Pearson correlation coefficient. Figure S1: different patterns were used to predict the prognosis. Figure S2: the ICI score predicts the prognosis and the GO analysis. Figure S3: the mutation genes in different ICI score groups and the prognosis of NRF2. Figure S4: the prognostic value of NRF2 and activated mast cells.
