# Supplementary material for: Activated Mast Cells Combined with NRF2 Predict Prognosis for Esophageal Cancer
Source: J Oncol. 2023 Jan 4;2023:4211885. doi: 10.1155/2023/4211885 (PMC9833916; doi:10.1155/2023/4211885)
Supplement: Supplementary Materials — Table S1: the primer sequences of related genes. Table S2: the DEGs in ICI gene signatures A and B. Table S3: association with overall survival and clinicopathological characteristics in patients from the TCGA database using Cox regression. Table S4: correlation of NRF2 with immune cells in patients from the TCGA database and GEO database using the Pearson correlation coefficient. Figure S1: different patterns were used to predict the prognosis. Figure S2: the ICI score predicts the prognosis and the GO analysis. Figure S3: the mutation genes in different ICI score groups and the prognosis of NRF2. Figure S4: the prognostic value of NRF2 and activated mast cells. [file 4211885.f1.zip › Table S4.doc]

Table S4. Correlation of NRF2 with immune cells in patients from the TCGA database and GEO database using Pearson correlation coefficient.

| Immune cell | r | *p*-value |
| --- | --- | --- |
| B cells naive | 0.071 | 0.263 |
| B cells memory | 0.062 | 0.332 |
| Plasma cells | -0.032 | 0.614 |
| T cells CD8 | -0.054 | 0.393 |
| T cells CD4 naive | 0.016 | 0.800 |
| T cells CD4 memory resting | 0.003 | 0.968 |
| T cells CD4 memory activated | 0.013 | 0.839 |
| T cells follicular helper | 0.017 | 0.787 |
| T cells regulatory (Tregs) | -0.065 | 0.310 |
| T cells gamma delta | 0.036 | 0.575 |
| NK cells resting | -0.088 | 0.164 |
| NK cells activated | 0.057 | 0.371 |
| Monocytes | -0.012 | 0.847 |
| Macrophages M0 | -0.092 | 0.149 |
| Macrophages M1 | -0.052 | 0.415 |
| Macrophages M2 | 0.061 | 0.339 |
| Dendritic cells resting | 0.117 | 0.064 |
| Dendritic cells activated | 0.030 | 0.639 |
| Mast cells resting | 0.125 | 0.049* |
| Mast cells activated | -0.150 | 0.018* |
| Eosinophils | 0.060 | 0.346 |
| Neutrophils | 0.066 | 0.066 |
