# Supplementary material for: Activated Mast Cells Combined with NRF2 Predict Prognosis for Esophageal Cancer
Source: J Oncol. 2023 Jan 4;2023:4211885. doi: 10.1155/2023/4211885 (PMC9833916; doi:10.1155/2023/4211885)
Supplement: Supplementary Materials — Table S1: the primer sequences of related genes. Table S2: the DEGs in ICI gene signatures A and B. Table S3: association with overall survival and clinicopathological characteristics in patients from the TCGA database using Cox regression. Table S4: correlation of NRF2 with immune cells in patients from the TCGA database and GEO database using the Pearson correlation coefficient. Figure S1: different patterns were used to predict the prognosis. Figure S2: the ICI score predicts the prognosis and the GO analysis. Figure S3: the mutation genes in different ICI score groups and the prognosis of NRF2. Figure S4: the prognostic value of NRF2 and activated mast cells. [file 4211885.f1.zip › FigureS4 (1).doc]

A

B

C

D

E

F

G

Figure S4. (A) overall survival curves of NRF2, (B) overall survival curves of FCER1G, (C) overall survival curves of combination NRF2 and FCER1G (D) expression of FCER1A, (E) expression of PD-L1, (F) relationship between PD-L1 and FCER1G; (G)relationship between NRF2 and FCER1G.
