# Supplementary material for: Activated Mast Cells Combined with NRF2 Predict Prognosis for Esophageal Cancer
Source: J Oncol. 2023 Jan 4;2023:4211885. doi: 10.1155/2023/4211885 (PMC9833916; doi:10.1155/2023/4211885)
Supplement: Supplementary Materials — Table S1: the primer sequences of related genes. Table S2: the DEGs in ICI gene signatures A and B. Table S3: association with overall survival and clinicopathological characteristics in patients from the TCGA database using Cox regression. Table S4: correlation of NRF2 with immune cells in patients from the TCGA database and GEO database using the Pearson correlation coefficient. Figure S1: different patterns were used to predict the prognosis. Figure S2: the ICI score predicts the prognosis and the GO analysis. Figure S3: the mutation genes in different ICI score groups and the prognosis of NRF2. Figure S4: the prognostic value of NRF2 and activated mast cells. [file 4211885.f1.zip › Table S1 (1).doc]

Table S1 The primer sequence of related genes

| gene | Forward primer | Reverse primer |
| --- | --- | --- |
| FcεR1A | CTTCTCACGCGGAGCTTTTATT | GGCAGCTGGACTATGAGTCTGA |
| NRF2 | CACATCCAGTCAGAAACCAGTGG | GGAATGTCTGCGCCAAAAGCTG |
| FcεR1G | GATGCCATCCTGTTTCTGTATGG | CACTTGGATCTTCAGTCGACAGTAG |
| PD-L1 | TGGATCCAGTCACCTCTGAAT | TGCTTGTCCAGATGACTTCG |
| GAPDH | TCGACAGTCAGCCGCATCTTCTTT | CCAAATCCGTTGACTCCGACCTT |
