# Supplementary material for: Activated Mast Cells Combined with NRF2 Predict Prognosis for Esophageal Cancer
Source: J Oncol. 2023 Jan 4;2023:4211885. doi: 10.1155/2023/4211885 (PMC9833916; doi:10.1155/2023/4211885)
Supplement: Supplementary Materials — Table S1: the primer sequences of related genes. Table S2: the DEGs in ICI gene signatures A and B. Table S3: association with overall survival and clinicopathological characteristics in patients from the TCGA database using Cox regression. Table S4: correlation of NRF2 with immune cells in patients from the TCGA database and GEO database using the Pearson correlation coefficient. Figure S1: different patterns were used to predict the prognosis. Figure S2: the ICI score predicts the prognosis and the GO analysis. Figure S3: the mutation genes in different ICI score groups and the prognosis of NRF2. Figure S4: the prognostic value of NRF2 and activated mast cells. [file 4211885.f1.zip › Table S2 (1).doc]

Table S2 The DEGs in ICI gene signature A and B

| signature A | signature B |
| --- | --- |
| MMP9  KLRB1  CPA3  ADH1C  CHI3L1  TNFRSF17  HSD17B2  IL33  IGLL5  CEACAM7  IDO1  GZMA  GZMB  GBP2  GZMH  CD8A  PSMB9  NKG7  CD2  GBP4  GBP1  RTP4  HLA-DPA1  CD3D  FGL2  BATF2  GBP5  ACE2  TRIM22  HLA-DRA  FOXA1  AIM2  IFI44L  ACSL5  CD38  IFIT1  BST2  CXCL17  CLCA4  C1QB  C1QA  IFI6  IFI27 | SPP1; INHBA; PXDN; COL6A1; COL6A3; PCOLCE; COL5A2; COL1A2; COL5A1; ZNF469; COL3A1; PMEPA1; FN1; COL12A1; POSTN; LAMC2; CSPG4; APCDD1L; MMP1; CXCL5; KANK4; PRAME; SHISA2; LCE3D; SPRR2G; MAGEA12 |
