# Supplementary material for: Activated Mast Cells Combined with NRF2 Predict Prognosis for Esophageal Cancer
Source: J Oncol. 2023 Jan 4;2023:4211885. doi: 10.1155/2023/4211885 (PMC9833916; doi:10.1155/2023/4211885)
Supplement: Supplementary Materials — Table S1: the primer sequences of related genes. Table S2: the DEGs in ICI gene signatures A and B. Table S3: association with overall survival and clinicopathological characteristics in patients from the TCGA database using Cox regression. Table S4: correlation of NRF2 with immune cells in patients from the TCGA database and GEO database using the Pearson correlation coefficient. Figure S1: different patterns were used to predict the prognosis. Figure S2: the ICI score predicts the prognosis and the GO analysis. Figure S3: the mutation genes in different ICI score groups and the prognosis of NRF2. Figure S4: the prognostic value of NRF2 and activated mast cells. [file 4211885.f1.zip › Table S3 (2).doc]

Table S3 Association with overall survival and clinicopathological characteristics in patients from the TCGA database using Cox regression.

| Variables  ID | Univariate Cox Regression | | | | Multivariate Cox Regression | | | |
| --- | --- | --- | --- | --- | --- | --- | --- | --- |
|  | HR | HR.95L | HR.95H | *p*-value | HR | HR.95L | HR.95H | *p*-value |
| Age | 0.66 | 0.676 | 1.687 | 0.777 | 0.300 | 0.720 | 2.529 | 0.349 |
| Gender | -0.714 | 0.994 | 0.212 | 1.130 | -1.109 | 0.077 | 1.449 | 0.143 |
| Grage | 0.599 | 1.079 | 3.072 | 0.025* | -0.332 | 0.359 | 1.431 | 0.346 |
| Stage | 1.235 | 1.989 | 5.941 | <0.001* | 0.521 | 0.510 | 5.563 | 0.393 |
| T | 0.452 | 0.941 | 2.626 | 0.084 | 0.675 | 0.788 | 4.892 | 0.147 |
| M | 1.043 | 1.624 | 4.954 | <0.001* | 0.448 | 0.525 | 4.664 | 0.421 |
| N | 1.018 | 1.562 | 4.901 | <0.001* | 0.756 | 0.763 | 5.938 | 0.149 |
| NRF2 | 0.055 | 0.379 | 2.946 | 0.916 | -0.332 | 0.159 | 3.249 | 0.667 |
